# Self-Paced Free-Running Wheel Mimics High-Intensity Interval Training Impact on Rats’ Functional, Physiological, Biochemical, and Morphological Features

**DOI:** 10.3389/fphys.2019.00593

**Published:** 2019-05-14

**Authors:** Jorge Beleza, João Albuquerque, Estela Santos-Alves, Pedro Fonseca, Garoa Santocildes, Jelena Stevanovic, Sílvia Rocha-Rodrigues, David Rizo-Roca, António Ascensão, Joan Ramon Torrella, José Magalhães

**Affiliations:** ^1^Laboratory of Metabolism and Exercise (LaMetEx), Department of Sport Biology, Faculty of Sport, Research Centre in Physical Activity, Health and Leisure (CIAFEL), University of Porto, Porto, Portugal; ^2^Porto Biomechanics Laboratory (LABIOMEP), University of Porto, Porto, Portugal; ^3^Departament de Biologia Cel ⋅ lular, de Fisiologia i d’Immunologia, Facultat de Biologia, Universitat de Barcelona, Barcelona, Spain

**Keywords:** voluntary physical activity, animals, skeletal muscle, mitochondria, heart

## Abstract

Free-running wheel (FRW) is an animal exercise model that relies on high-intensity interval moments interspersed with low-intensity or pauses apparently similar to those performed in high-intensity interval training (HIIT). Therefore, this study, conducted over a 12-weeks period, aimed to compare functional, thermographic, biochemical and morphological skeletal and cardiac muscle adaptations induced by FRW and HIIT. Twenty-four male Wistar rats were assigned into three groups: sedentary rats (SED), rats that voluntarily exercise in free wheels (FRW) and rats submitted to a daily HIIT. Functional tests revealed that compared to SED both FRW and HIIT increased the ability to perform maximal workload tests (MWT-cm/s) (45 ± 1 vs. 55 ± 2 and vs. 65 ± 2). Regarding thermographic assays, FRW and HIIT increased the ability to lose heat through the tail during MWT. Histochemical analyzes performed in *tibialis anterior* (TA) and *soleus* (SOL) muscles showed a general adaptation toward a more oxidative phenotype in both FRW and HIIT. Exercise increased the percentage of fast oxidative glycolytic (FOG) in medial fields of TA (29.7 ± 2.3 vs. 44.9 ± 4.4 and vs. 45.2 ± 5.3) and slow oxidative (SO) in SOL (73.4 ± 5.7 vs. 99.5 ± 0.5 and vs. 96.4 ± 1.2). HITT decreased fiber cross-sectional area (FCSA-μm^2^) of SO (4350 ± 286.9 vs. 4893 ± 325 and vs. 3621 ± 237.3) in SOL. Fast glycolytic fibers were bigger across all the TA muscle in FRW and HIIT groups. The FCSA decrease in FOG fibers was accompanied by a circularity decrease of SO from SOL fibers (0.840 ± 0.005 vs. 0.783 ± 0.016 and vs. 0.788 ± 0.010), and a fiber and global field capillarization increase in both FRW and HIIT protocols. Moreover, FRW and HIIT animals exhibited increased cardiac mitochondrial respiratory control ratio with complex I-driven substrates (3.89 ± 0.14 vs. 5.20 ± 0.25 and vs. 5.42 ± 0.37). Data suggest that FRW induces significant functional, physiological, and biochemical adaptations similar to those obtained under an intermittent forced exercise regimen, such as HIIT.

## Introduction

The use of animal models is a common practice in exercise-based studies. In fact, exercise physiologists use different animal models, in particular rodents, to study the physiological and biochemical impact of exercise induced by forced running endurance training in treadmill, swimming, or climbing stairs [for a complete guide of training methods and animal models for the study of exercise see Kregel et al. (2006)]. Moreover, voluntary self-paced motor behavior models, such as 24-h access to free-running wheels (FRW), are used to minimize (mal)adaptations associated to sedentarism or pathological conditions (Fonseca et al., 2011a; Goncalves et al., 2014). Having animals usually housed in small cages, in this FRW model, wheels are introduced in the housing cages and animals are allowed to freely run at chosen intensities and durations (Goncalves et al., 2014, 2016; Marques-Aleixo et al., 2016). However, despite the widespread use of FRW, there is a lack of knowledge regarding the truly physiological and biochemical impact of this model on voluntarily exercised animals. Moreover, when considering the motor pattern, this model is characterized by short or middle-duration bouts of exercise performed at high speed interspersed by long-lasting pauses and periods of low intensity that, in certain sense, seem to mimic the exercise bouts performed during sessions of high-intensity interval training (HIIT) (Novak et al., 2012).

Recently introduced as an exercise model, HIIT has proved to be a powerful stimulus to induce rapid skeletal muscle remodeling and to enhance skeletal muscle oxidative capacity in humans (Ramos-Filho et al., 2015). Furthermore, HIIT seems to reduce the rate of lactate production during exercise, to increase the capacity of skeletal muscle lipid oxidation (Gibala and Jones, 2013), and to induce an increased post-exercise metabolic rate (Schaun et al., 2017). However, despite the increasing use of HIIT in animal studies (Ramos-Filho et al., 2015; Delwing-de Lima et al., 2017; Freitas et al., 2018; Shirvani and Arabzadeh, 2018), data regarding its overall exercise intensity and metabolic impact is still scarce. Therefore, considering distinct levels of tissue and cellular organization, we here aim to analyze the different skeletal and cardiac muscles response induced by FRW and HIIT. For skeletal muscle, histomorphological features using two different phenotypic specimens, mainly oxidative *soleus* (SOL) and the *tibialis anterior* (TA), will be analyzed. For cardiac muscle, mitochondrial function will be used as an important hallmark of cardiac tissue metabolic fitness and adaptation (Ascensao et al., 2006; Magalhaes et al., 2013, 2014), and bioenergetics endpoints associated with *in vitro* oxygen consumption will be obtained. Moreover, taking in to account that energy expenditure in rats seems to be related with alterations in body temperature (Wanner et al., 2015b), namely in skin temperature (Wanner et al., 2015a), measurements on the animals’ tail temperature (*T*_tail_) during maximal workload tests will also be performed.

To the best of our knowledge, this is the first study that compares, from a physiological, biochemical, and morphological point of view, the impact of both FRW and HIIT exercise regimens in a rodent model. Taken together, data will contribute to better understand the impact of these often-used exercise models on skeletal and cardiac muscle and to truly clarify the relevance of the FRW when compared to other more formal and forced exercise animal models, such as HIIT.

## Materials and Methods

### Animals

All experimental procedures involving animals were performed in accordance with guidelines for Care and Use of Laboratory Animals in Research advised by Federation of European Laboratory Animal Science Associations (FELASA). The Ethics Committee of the Faculty of Sport, University of Porto and the National Government Authority (*Direção Geral de Alimentação e Veterinánia* – N^*o*^ 0421/000/000/2018) approved the experimental protocol.

Wistar male rats (*n* = 24) aged 7–8 weeks old were obtained from Charles River Laboratories (L’Arbresle, France) and were housed in individual cages under controlled environmental conditions (21–22°C; 50–60% humidity), receiving standard laboratory pellet chow (4RF21, Mucedola, Italy) and water *ad libitum* in 12-h light/dark cycles. After 1 week of quarantine, animals were randomly assigned into three groups (*n* = 8/group): sedentary (SED), FRW, and HIIT.

### Maximal Workload Test (MWT) and VO_2max_ Assessment

In the context of the present study, MWT were performed both prior to the training/FRW and at the end of the protocol, being VO_2max_ only evaluated in the referred first moment. For these purposes, animals were adapted to a motorized treadmill for 5 days (LE8700, Panlab, Harvard, United States). In the first day, rats were placed on the immobile treadmill to adapt to the new device and in the second day, the treadmill was turned on and the speed and duration were gradually increased during the subsequent 4 days. Moreover, electric-based shock grids were used to stimulate rats to run.

In the MWT, the animals were individually evaluated on the treadmill at an inclination of 15°. The test started at a speed of 15 cm/s, after 5 min the speed was increased to 30 cm/s and then stepped up 3 cm/s every 2 min until maximal workload was achieved. This MWT allowed to achieve VO_2max_-related speed in order to set the different workloads for the HIIT protocol described below, being VO_2max_ evaluated on a close chamber treadmill coupled to a gas analyzer (LE405 Gas Analyser, Panlab, Barcelona, Spain). Two criteria were established to confirm maximal workload, namely the exhaustion of the animal and blood lactate concentration higher than 7 mmol/L. Immediately after the end of MWT and in different time points (1, 3 and 5 min after), blood samples were collected from the animals’ tail until a maximal value of lactate concentration (Lactate Plus Meter, Nova Biomedical, United States) was achieved. However, to confirm the achievement of VO_2max_, two additional criteria were considered, namely the plateau in oxygen consumption and respiratory coefficient higher than 1.

### Exercise Protocols

Animals from the FRW group were housed in a polyethylene cage equipped with a running wheel (circumference = 1.05, Type 304 Stainless steel, Tecniplast, Casale Litta, Italy). The rats had free access to the running wheel 24 h/day and the running distance was recorded using a digital counter (ECO 701 Hengstler, Lancashire, United Kingdom).

**Table 1:** Summary of speed achieved during the experimental period.

	Week												
	1	2	3	4	5	6	7	8	9	10	11	12	
Warm up	33	33	33	33	33	33	33	35	35	35	35	35	Speed (cm ⋅ s^−1^)
Bout 1	45	45	45	45	45	45	45	47	47	47	47	47	
Bout 2	46	46	46	46	47	47	47	49	49	49	49	49	
Bout 3	47	47	47	47	49	49	49	51	51	51	51	51	
Bout 4	48	48	48	48	51	51	51	53	53	53	53	53	

Animals from the HIIT group were exercised during 12 weeks (5 days/week), Monday to Friday, between 10:00 and 12:00 AM, on the motor treadmill at 15° inclination. The HIIT protocol started with 5 min warm up at low speed correspondent to 60% of VO_2max_, then the rats performed four bouts of 4 min at speeds correspondent to 85–90% of VO_2max_ interspersed by 2 min of active recovery at speed correspondent to 60% of VO_2max_ [adapted from Songstad et al. (2015)]. Nevertheless, during training sessions the speed was gradually increased to meet animals’ adaptations throughout the protocol (Table 1) and electric-based shock grids were used to stimulate rats to run. Both SED and FRW animals were placed on a non-moving treadmill to be exposed to the same handling and environmental conditions.

### Thermographic Measurements

**FIGURE 1:**
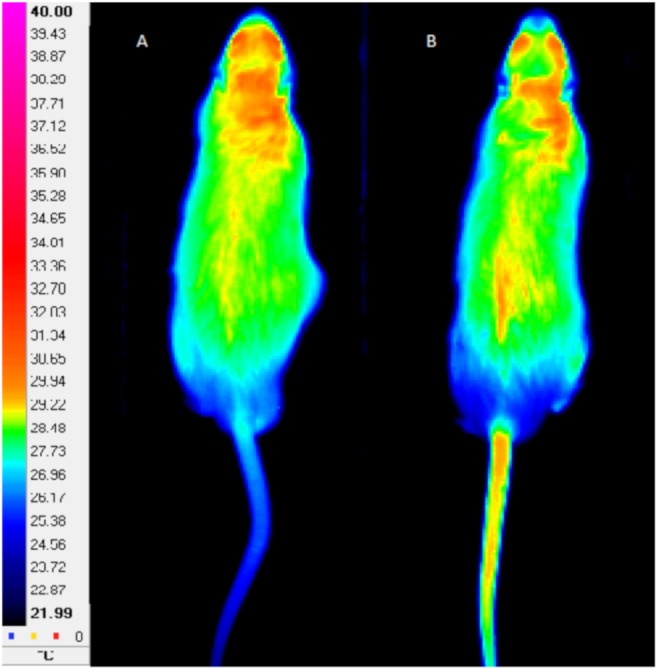
Example of tail thermographs obtained at the beginning **(A)** and at the end **(B)** of maximal workload test. Left scale indicates a color code for temperature in °C.

A thermographic camera (FLIR A325, FLIR Systems, Wilsonville, United States) properly calibrated, presenting the resolution of 320 × 240 dpi, sensitivity of 70 mK and accuracy of ±2%, was used for the acquirement of animal tail thermographs. The distance between the camera and the photographed animal was set at 2 m. In each measurement, the assessment of the *T*_tail_ was obtained, by the average of the temperature in the whole tail during 5 s (Figure 1). The analysis was performed using the software FLIR ThermaCAM Researcher Pro 2.10^®^. Thermographs were obtained in the start and at the end of the 12 weeks of exercise regimens (FRW and HIIT), at the beginning (Figure 1A) and in each step until the end of the MWT (Figure 1B).

### Muscle Sampling and Histochemical Procedures

All animals were fasted overnight for 12 h with access to drinking water before sacrifice. Animals were weighed at the day of sacrifice and anesthetized with ketamine (90 mg ⋅ kg^−1^) and xylazine (10 mg ⋅ kg^−1^). Right SOL and TA muscles were excised, immediately frozen in pre-cooled isopentane and stored at −80°C until further analysis.

Muscle samples were mounted in embedding medium (Tissue-Tek^®^, Sakura Finitek Europe, Zoeterwoude, Netherlands), and cut in serial transverse sections (12–16 μm) using a cryostat (Leica CM3050S, Wetzlar, Germany) at −22°C. Sections were mounted on gelatinized (0.02%) slides and stained for: (1) myofibrillar adenosine triphosphatase (mATPase) following pre-incubation in alkaline (pH 10.7) and acid (pH 4.2) mediums to differentiate between slow and fast-twitch fibers (Brooke and Kaiser, 1970); (2) endothelial adenosine triphosphatase (eATPase) to reveal muscle capillaries (Fouces et al., 1993); and (3) succinate dehydrogenase (SDH) to distinguish between aerobic and anaerobic fibers (Nachlas et al., 1957).

### Morphofunctional Measurements

**FIGURE 2:**
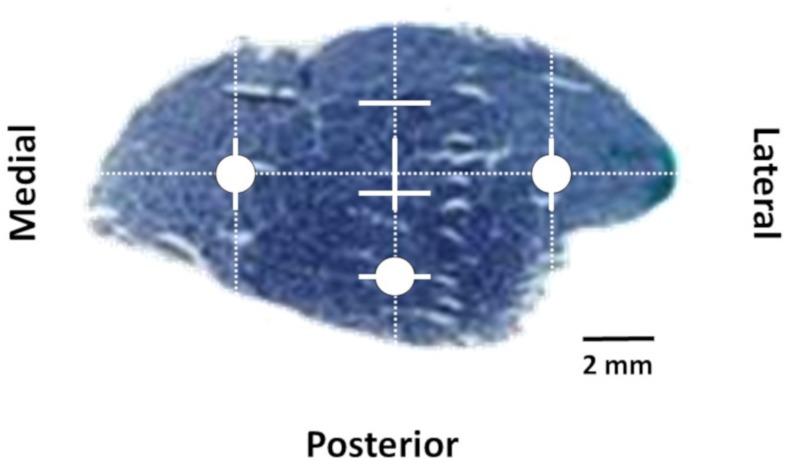
Equatorial transverse section of *tibialis anterior*. Circles indicates lateral, medial, and posterior zones selected for histochemical and morphofunctional analyses.

Microphotographs obtained with a light microscope (BX61, Olympus, Tokyo, Japan) connected to a digital camera (DP70, Olympus, Tokyo, Japan) at a magnification of 100x were used to obtain muscle fiber morphofunctional measurements. The analyzed parameters were obtained from transverse cross sections of approximately 5.5 × 10^5^ μm^2^ from three different microphotographs and were treated using an image analyzing software (ImageJ; Rasband 1997–2014). The TA was analyzed following the protocol described in Torrella et al. (2000), represented schematically in Figure 2. First, we chose the major axis of the muscle transverse section and divided its total length into four equal intervals. Oriented from medial to lateral, the end of the first interval was chosen as medial zone whilst the beginning of the fourth interval was chosen as lateral zone. Subsequently, a secondary orthogonal axis was drawn to cross the central division of the major longitudinal axis. This orthogonal axis was also divided in four equal intervals. Oriented from anterior to posterior, the beginning of the fourth interval was chosen as posterior zone. An image of the equatorial zone of the muscle section was obtained using a light-stereoscope (Olympus, SZ40, Japan) and the sections were divided in a grid-like structure throughout two-dimensional axis to provide the sample areas (or fields) to be studied for each anatomical region (medial, lateral, and posterior). Due to their different fiber type composition and morphometry, data are presented separately for each of these different regions.

**FIGURE 3:**
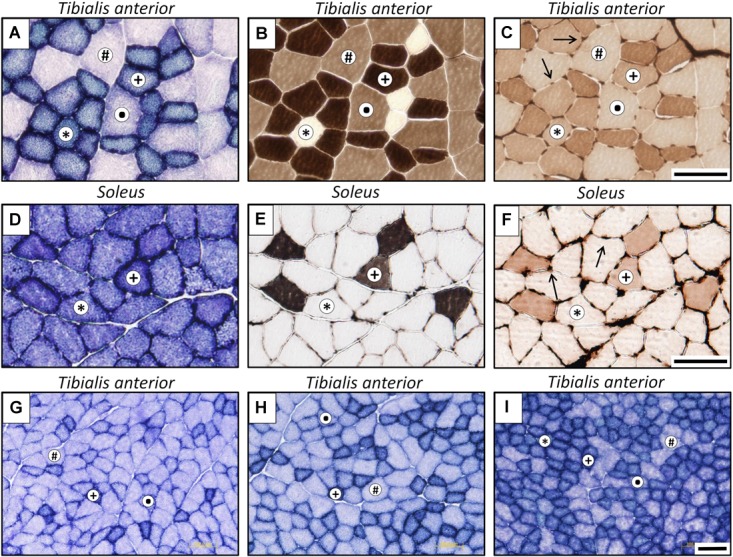
Representative microphotographs of muscle cross-sections stained for different histochemical assays: succinate dehydrogenase **(A,D,G–I)**, myosin ATPase after alkaline pre-incubation **(B,E)**, and endothelial ATPase (**C,F,** arrows indicate muscle capillaries). Superior panel: posterior zone of *tibialis* anterior muscle; middle panel: soleus muscle; lower panel: three different zones at lower magnification of the *tibialis* anterior muscle (**G**: lateral; **H**: medial; **I**, posterior). Fiber types are identified with symbols: ^∗^, slow oxidative (SO); +, fast oxidative glycolytic (FOG);, fast intermediate glycolytic (FIG); #, fast glycolytic (FG). Scale bar, 100 μm.

Fibers were typified (Figure 3) as slow oxidative (SO), fast oxidative glycolytic (FOG), fast glycolytic (FG) or fast intermediate glycolytic (FIG), with higher SDH staining than FG, but lower staining than FOG fibers.

The fiber cross-sectional area (FCSA) and the shape factor circularity (SF = 4 ⋅ π FCSA/perimeter^2^) were measured. The following parameters related with fiber capillarization were also obtained: the absolute number of capillaries per fiber (NCF), the relative the number of capillaries per 1,000 μm^2^ of FCSA (CCA), fiber density (FD, mm^−1^), capillary density (CD, mm^−1^), and capillary-to-fiber ratio (C/F = CD/FD).

### Isolation of Heart Mitochondria

Mitochondria from left heart ventricles were isolated using conventional methods of differential centrifugation as described elsewhere (Bhattacharya et al., 1991). Mitochondrial and homogenate protein contents were determined by the Biuret method calibrated with BSA (Van Norman, 1909). All mitochondrial isolation procedures were performed at 0–4°C. Considering the relatively greater abundance of intermyofibrillar (IMF) (∼80%) compared with subsarcolemmal (SS) (∼20%) mitochondria within the cells, a potentially dominant role for the IMF subfraction vs. the SS subfraction when studying mitochondrial alterations is expected.

### Mitochondrial Oxygen Consumption Assays

Mitochondrial respiratory function was polarographically measured at 30°C, using a Biological Oxygen Monitor system (Hansatech Instruments, Norfolk, United Kingdom) and a Clark-type oxygen electrode (Hansatech DW 1, Norfolk, United Kingdom). Reactions were conducted in a 0.75 mL closed glass chamber, thermostated and magnetically stirred containing 0.5 mg of mitochondrial protein in a reaction buffer with 65 mM KCl, 125 mM sucrose, 10 mM Tris, 20 mM EGTA, 2.5 mM KH2PO4, pH 7.4. After 1-min equilibration period, mitochondrial respiration was initiated by adding glutamate (10 mM) plus malate (5 mM) as substrates for complex I or succinate (10 mM) plus rotenone (4 mM) as complex II-related substrates.

State 3 respiration (oxygen consumption after adding 125 nmol ADP) and state 4 (considered as the rate of oxygen after full ADP phosphorylation) were measured. The respiratory control ratio (RCR, state 3/state 4) and the ADP/O ratio (nmol ADP phosphorylated by natom O consumed), were calculated according to Estabrook (1967), using 474 natom O mL^−1^ as the value of solubility of oxygen at 25°C in double-distilled water.

### Mitochondrial Membrane Potential Measurements

Mitochondrial transmembrane potential (Δψ) was indirectly measured based on the activity of the lipophilic cation tetraphenylphosphonium (TPP^+^), through a TPP^+^ selective electrode previously prepared as described by Lumini-Oliveira et al. (2011). Reactions were carried out in 1 mL of reaction buffer containing 65 mM KCl, 125 mM sucrose, 10 mM Tris, 20 mM EGTA, 2.5 mM KH_2_PO_4_, pH 7.4, supplemented with 3 μM TPP^+^, and 0.5 mg/mL of protein at the temperature kept at 25°C. For measurements of Δψ with complex I substrates, energization was carried out with 10 mM of glutamate and 5 mM of malate and ADP-induced phosphorylation was accomplished by adding 125 nmol ADP. For measurements of Δψ with complex II substrates, 10 mM succinate supplemented with 4 μM rotenone were added to the medium containing 3 mM TPP^+^ and mitochondria. The lag phase, which reflects the time required to phosphorylate the added ADP, was also measured for both substrates.

### Citrate Synthase Activity in Skeletal Muscle

Citrate synthase (CS) activity was measured in SOL and TA homogenates, as a marker of mitochondrial content and oxidative capacity, using the method described by Srere (1969). Briefly, 10 mg of muscle were homogenized in 100 mL of ice-cold medium containing 75 mM Tris ⋅ HCl, 2 mM MgCl_2_, and 1 mM EDTA (pH 7.6). The CoA-SH released from the reaction of acetyl-CoA with oxaloacetate was measured by its reaction with a colorimetric agent [5, 5-dithiobis (2-nitrobenzoate)]. The enzymatic activity was measured spectrophotometrically at 412 nm.

### Statistical Analysis

Data are reported as the mean ± SEM (standard error of the mean). Statistical analyses were performed using GraphPad Prism (version 7.0). One-way analysis of variance (ANOVA) was followed by Bonferroni *post hoc* test to compare differences between groups. In all cases, a value of *p* < 0.05 was considered statistically significant.

**Table 2:** Final body weight and muscle to body weight ratio.

Group	Body Weight (g)	*Soleus*/Body Weight (mg/g)	*Tibialis* Anterior/Body Weight (mg/g)
SED	390 ± 28.11	2.24 ± 0.16	0.40 ± 0.04
FRW	411 ± 4.86	2.24 ± 0.16	0.53 ± 0.02
HIIT	354 ± 5.42^∗#^	1.95 ± 0.08	0.51 ± 0.05

*SED, sedentary; FRW, free-running wheel; HIIT, high-intensity interval training. Data are expressed as the mean ± SEM. Statistical significant differences (p ≤ 0.05) are indicated as follows: ^∗^ vs. SED, ^#^ vs. FRW.*

## Results

### Physical Exercise and Body Weight

**FIGURE 4:**
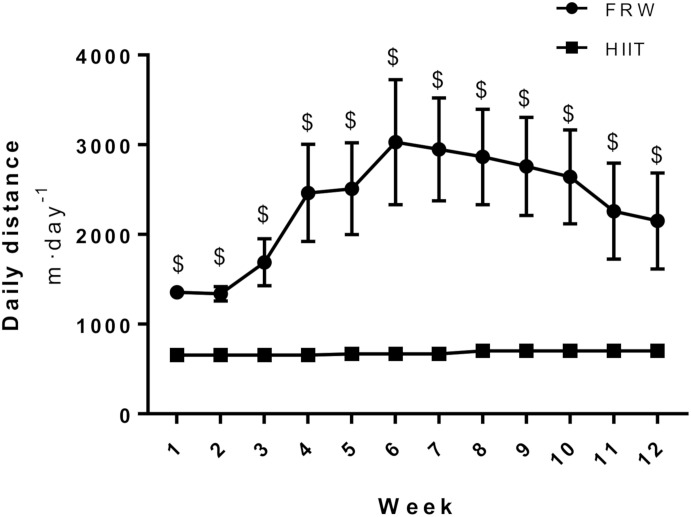
Mean distance covered per day. SED, sedentary; FRW, free-running wheel; HIIT, high-intensity interval training. Data are expressed as the mean ± SEM. Statistical significant differences (*p* ≤ 0.05) are indicated as follows: $ vs. HIIT.

After the 12 weeks of protocol, FRW animals ran a significantly higher overall day distance than HIIT (Figure 4). Their activity increased from the beginning of the protocol and reached a peak at the 6th week, when a non-significant decrease was observed until the end of the protocol. However, 12 weeks of HIIT decreased body weight, while FRW did not elicit significant alterations in this parameter (Table 2).

### Maximal Workload Test-Related Speed, Tail Temperature, and Blood Lactate Concentration

**FIGURE 5:**
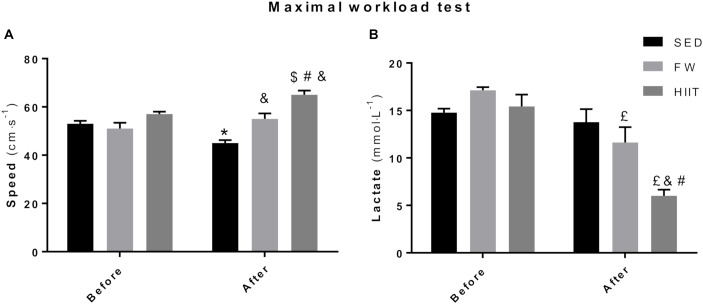
**(A)** Final speed achieved at the end of the maximal workload test before and after the experimental protocol. **(B)** Highest blood lactate concentration measured at the end of the maximal workload. SED, sedentary; FRW, free-running wheel; HIIT, high-intensity interval training. Data are expressed as the mean ± SEM. Statistical significant differences (*p* ≤ 0.05) are indicated as follows: ^∗^ vs. SED before, ^&^ vs. SED after, ^$^ vs. HIIT before, ^#^ vs. FRW after, £ vs. FRW before.

At the end of the 12 weeks, SED animals achieved a lower maximal speed in the MWT (from 57 to 48 cm/s) whereas both FRW (from 54 to 60 cm/s) and HIIT (from 57 to 69 cm/s) increased their maximal speed when compared to baseline (Figure 5A). No differences between groups in the maximal velocities at the beginning of the protocol were registered. However, significant differences were evident between groups at the end of the 12 weeks. In fact, HIIT and FRW animals reached significantly higher velocities compared to SED. Moreover, a significant difference was also found between HIIT and FRW (69 vs. 60 cm/s, respectively).

To find out how glycolytic energy pathway was modulated by this exercise programs and also in which comparative extent oxidative-related demands may support different exercise intensities and workloads after both FRW and HIIT, maximal blood lactate concentrations (BLC) were measured at the end of the MWT, before and after the 12 weeks of the protocol (Figure 5B). No significant differences were found at the beginning of the protocol in maximal BLC. However, FRW animals showed a lower BLC at the end of MWT after 12 weeks of free access to the running wheel (FRW: 11.63 ± 3.95 mmol/L vs. SED: 13.77 ± 3.35 mmol/L). Moreover, the animals submitted to the HIIT regimen reached a lower BLC at the end of the MWT (HIIT: 6.00 ± 1.85 mmol/L vs. SED: 13.77 ± 3.35 mmol/L and FRW:11.63 ± 3.95 mmol/L), despite attained a higher maximal speed when compared to those of the SED and FRW groups.

**FIGURE 6:**
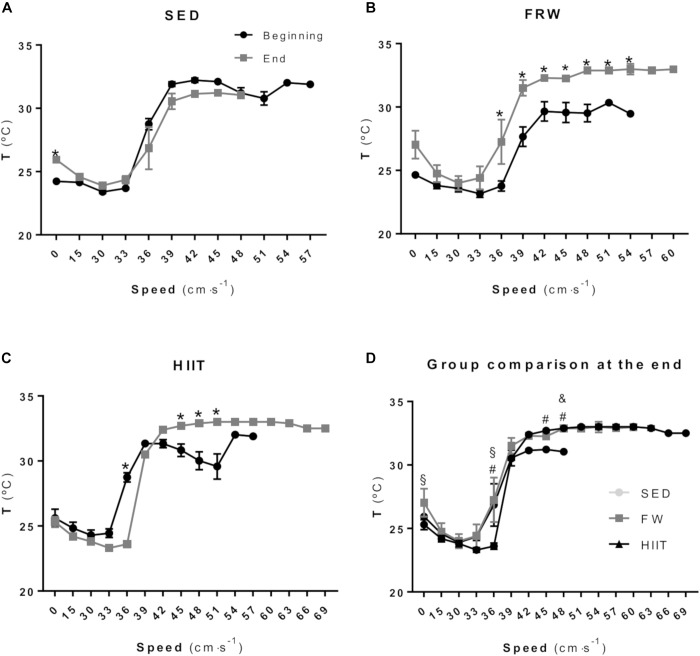
Tail temperature measured during the maximal workload test at the beginning and at the end of experimental protocols in SED **(A)**, FRW **(B)** and HIIT **(C)**. Comparison between the three groups of the tail temperature measured during maximal workload test at the end of experimental protocol **(D)**. SED, sedentary; FRW, free-running wheel; HIIT, high-intensity interval training. Data are expressed as the mean ± SEM. Statistical significant differences (*p* ≤ 0.05) are indicated as follows: ^∗^ beginning vs. end of the experimental protocol, ^&^ SED vs. FRW, ^#^ SED vs. HIIT, ^§^ FRW vs. HIIT.

Figure 6 shows the tail temperature (*T*_tail_) at the different steps of the MWT before and after the 12 weeks of the intervention protocol. SED animals did not show statistical differences in *T*_tail_ in any step, with exception of the baseline (Figure 6A). After the training period, FRW animals showed an increased T_tail_ from speeds 36 to 54 cm/s when compared to baseline (Figure 6B). The HIIT regimen induced a significantly decreased T_tail_ at 36 cm/s. However, at speeds ranging from 45 to 51 cm/s, significant increments were obtained when compared to baseline (Figure 6C). A comparison between groups at the end of the 12 weeks in resting conditions (Figure 6D) showed that HIIT animals presented a lower T_tail_ compared to FRW; compared to FRW and SED at 36 cm/s, but a higher T_tail_ at 45 and 48 cm/s when compared to SED. The FRW animals revealed a higher T_tail_ at 48 cm/s compared to SED.

### Citrate Synthase Activity

**FIGURE 7:**
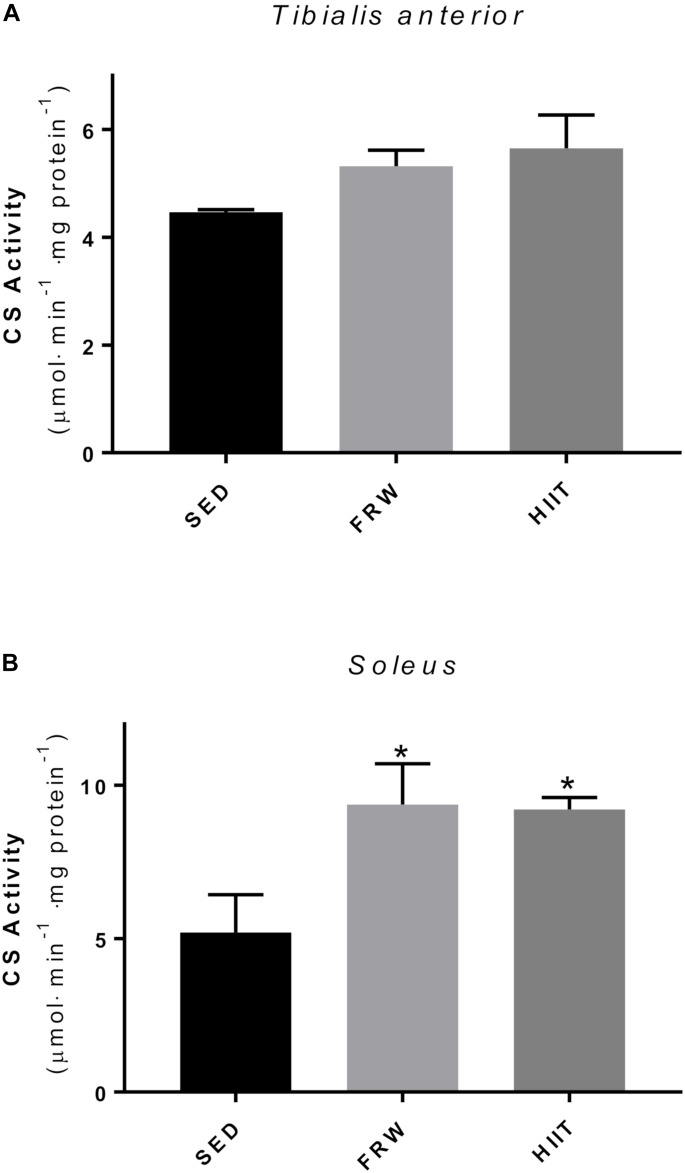
Effects of physical exercise on the citrate synthase activity (CS) of *Tibialis* anterior **(A)** and *Soleus* **(B)** muscles. SED, sedentary; FRW, free-running wheel; HIIT, high-intensity interval training. Data are expressed as the mean ± SEM. Statistical significant differences (*p* ≤ 0.05) are indicated as follows: ^∗^ vs. SED.

Figure 7 shows CS activity in TA and SOL muscles as a marker of mitochondrial oxidative capacity. No significant differences were observed on TA after both chronic exercise regimens (Figure 7A). The activity in the SOL muscle was significantly higher in both exercised groups compared to SED control (Figure 7B).

### Cardiac Mitochondrial Function

**FIGURE 8:**
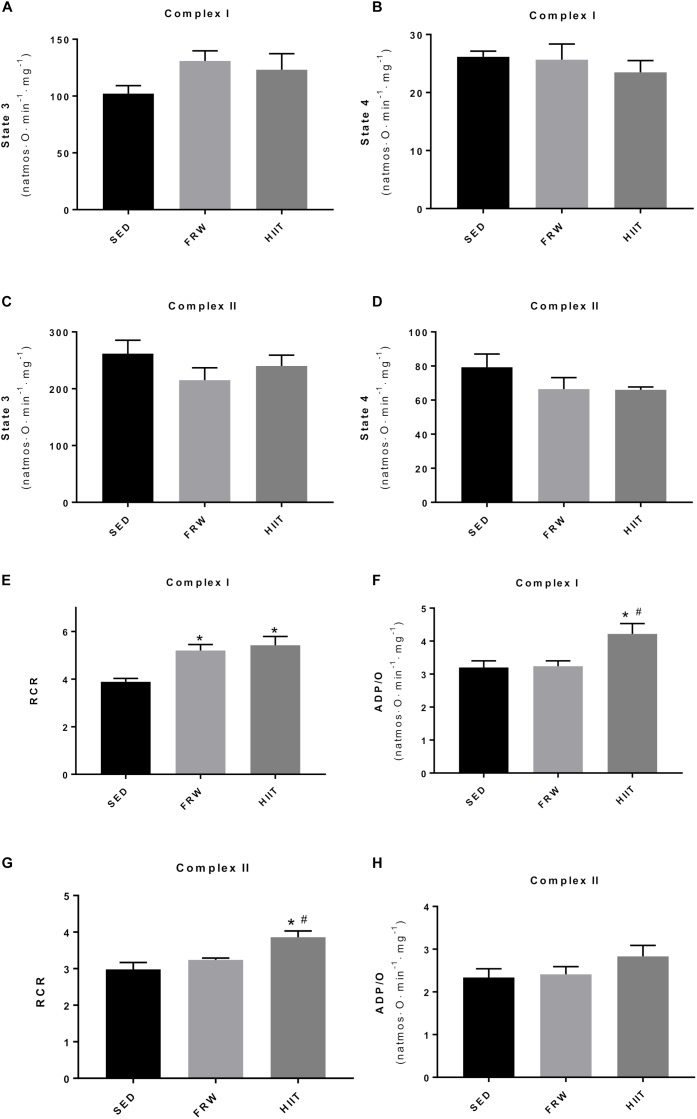
Effects of physical exercise on heart mitochondrial oxygen consumption measured with substrates for complex I and II. **(A,C)** state 3 respiration; **(B,D)** state 4 respiration; **(E,G)** RCR, respiratory control ratio (state 3/state 4); **(F,H)** ADP/O, number of nmol ADP phosphorylated by natom of oxygen consumed. SED, sedentary; FRW, free-running wheel; HIIT, high-intensity interval training. Data are expressed as the mean ± SEM. Statistical significant differences (*p* ≤ 0.05) are indicated as follows: ^∗^ vs. SED, ^#^ vs. FRW.

Despite no alterations were found in heart mitochondrial state 3 and 4 when complex I- and II-driven substrates were used after 12 weeks of both training modalities (Figure 8A–D), an increase in RCR with complex I-driven substrates (3.89 ± 0.14 vs. 5.20 ± 0.25 and 5.42 ± 0.37) in FRW and HIIT groups was evident (Figure 8E). When complex II-related substrates were used, HIIT induced an increase in RCR (3.86 ± 0.17 vs. 2.98 ± 0.19 and 3.24 ± 0.05) compared with SED and FRW conditions (Figure 8G). Phosphorylating efficiency, measured as ADP/O ratio, was higher in HIIT group (4.22 ± 0.31 vs. 3.20 ± 0.20 and 3.24 ± 0.16) for complex I-driven substrates (Figure 8F), while no alterations between groups were found when substrates for complex II were used (Figure 8H).

No alterations between groups were found in the transmembrane electrical potential endpoints (maximal potential, repolarization, and lag phase) for both complexes I- and II-related substrates (data not shown).

### Skeletal Muscle Fiber Type Distribution

**FIGURE 9:**
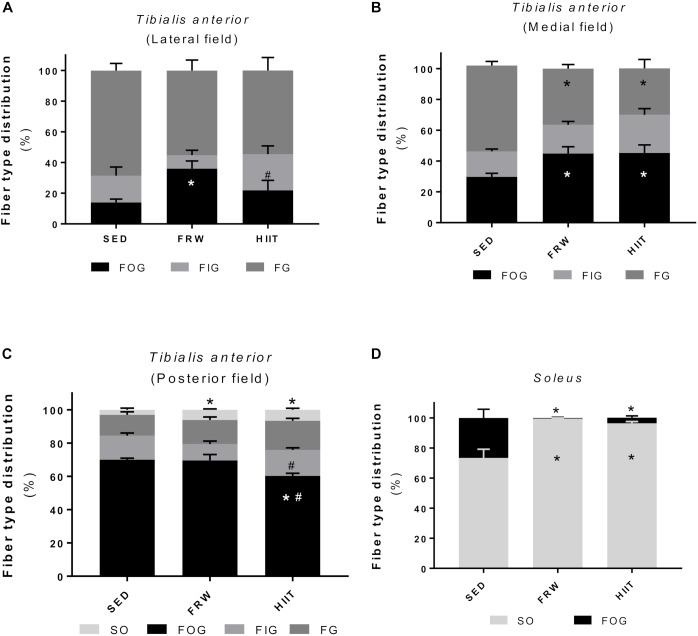
Effects of physical exercise on fiber type distribution in three *tibialis* anterior regions (**A**, lateral; **B**, medial; **C**, posterior) and in *soleus* muscle **(D)**. Fibers were classified as slow oxidative (SO), fast oxidative glycolytic (FOG), fast intermediate glycolytic (FIG), or fast glycolytic (FG). SED, sedentary; FRW, free-running wheel; HIIT, high-intensity interval training. Data are expressed as the mean ± SEM. Statistical significant differences (*p* ≤ 0.05) are indicated as follows: ^∗^ vs. SED, ^#^ vs. FRW.

Both chronic exercise models caused alterations in skeletal muscle fiber type distribution in TA and SOL (Figure 9). In the lateral field of TA, the FRW group exhibited an increased percentage of FOG fibers (36.0 ± 5.0 vs. 14.0 ± 2.2 and vs. 21.9 ± 6.5), while a significant increase in the percentage of FIG fibers was observed in the HIIT group (Figure 9A). Moreover, both FRW and HIIT increased the percentage of FOG fibers in the TA medial field (44.9 ± 4.4 and 45.2 ± 5.3 vs. 29.7 ± 2.3), which is concomitant with the decreased percentage of FG fibers (Figure 9B). Regarding SO fibers, only present in the posterior field, both FRW and HIIT exercise models induced an increased percentage (Figure 9C). Furthermore, in the SOL muscle, FRW, and HIIT elicited a decrease in the percentage of FOG fibers and a corresponding increment of SO fibers (Figure 9D).

### Skeletal Muscle Fiber Cross-Sectional Area and Fiber Shape

**FIGURE 10:**
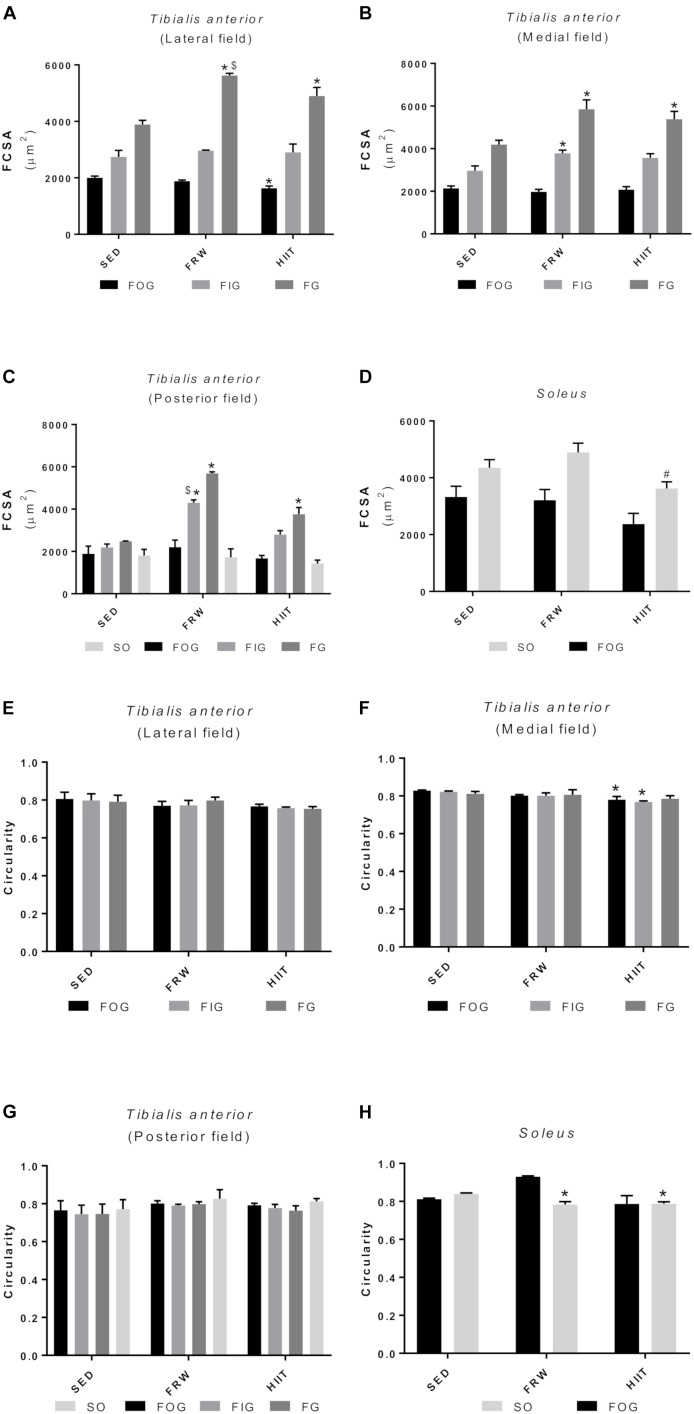
Effects of physical exercise on the mean fiber cross-sectional area **(A–D)** and fiber shape **(E–H)** in three *tibialis* anterior regions (**A**, lateral; **B**, medial; **C**, posterior) and in *soleus* muscle. SED, sedentary; FRW, free-running wheel; HIIT, high-intensity interval training. Data are expressed as the mean ± SEM. Statistical significant differences (*p* ≤ 0.05) are indicated as follows: ^∗^ vs. SED, ^#^ vs. FRW, ^$^ vs. HIIT.

Despite no evidence of changes in muscle to body weight ratio (Table 2), alterations in FCSA and fiber shape were registered in the present protocol. Figure 10 shows the FCSA from muscles TA (Figure 10A–C) and SOL (Figure 10D). In TA muscle, both FRW and HIIT induced a significant increase in FCSA of FG fibers in all the muscle regions. This increase was also evident in FIG fibers from the medial (3781.00 ± 146.07 vs. 2966 ± 222.86 and 3569.00 ± 193.41 μm^2^) and posterior fields (4301.00 ± 137.49 vs. 2192 ± 157.62 and 2796.00 ± 189.95 μm^2^) in FRW animals, but not evidenced after the HIIT protocol. Both oxidative fiber types (SO and FOG) showed no significant changes in FCSAs after any exercise protocol, except for FOG fibers from the lateral field of HIIT animals, in which a significant reduction in fiber size was observed (1634.00 ± 76.79 vs. 2001.00 ± 61.19 and 1881.00 ± 42.146 μm^2^). In SOL muscle, a significant reduction in FCSA was measured in SO fibers of HIIT animals (3621.00 ± 237.30 vs. 4350.00 ± 286.90 and 4893.00 ± 325.00 μm^2^).

Regarding the shape of the fibers (circularity), no significant alterations in the TA lateral and posterior fields were observed (Figure 10E–G). In contrast, a reduction in the circularity of the FOG and SO fibers HIIT animals was found in the TA medial field (Figure 10F). Furthermore, both exercise models decreased the circularity of SO fibers in the SOL (Figure 10H).

### Fiber Capillarization

**FIGURE 11:**
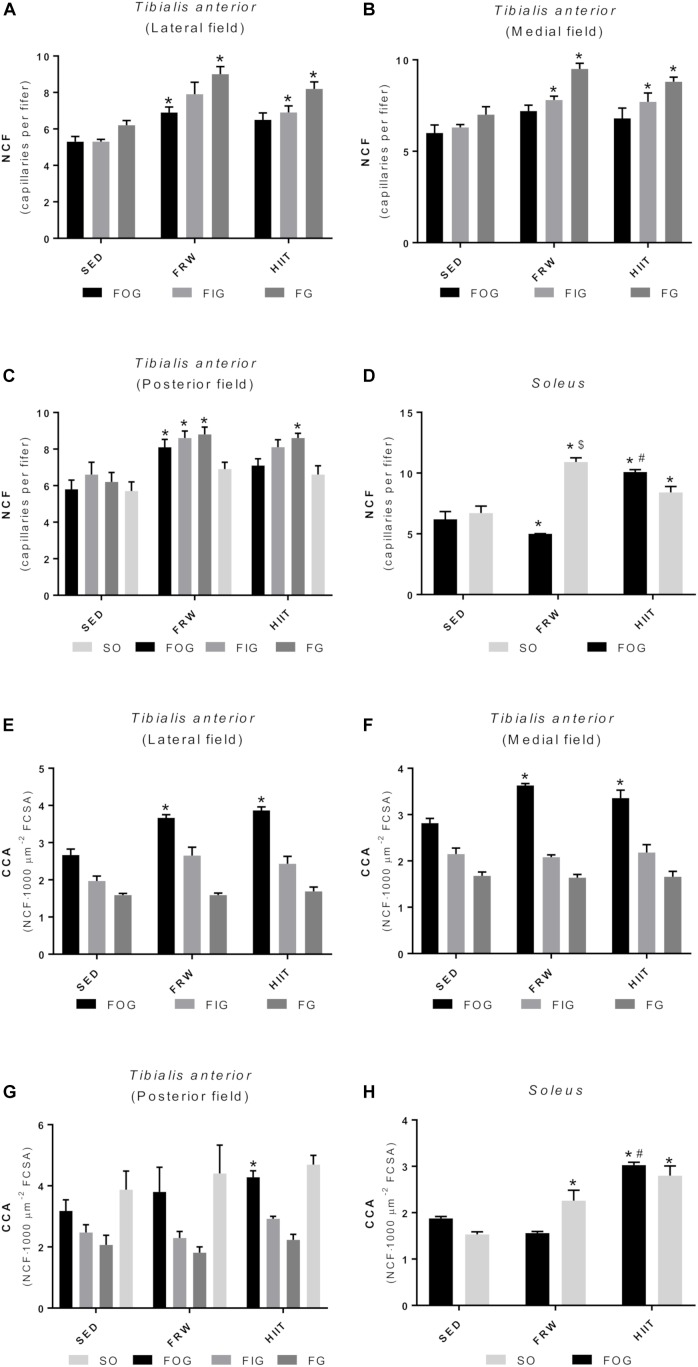
Effects of physical exercise on the NCF **(A–D)** and fiber capillarization index (CCA) **(E–H)** in three *tibialis* anterior regions (**A**, lateral; **B**, medial; **C**, posterior) and in *soleus* muscle. SED, sedentary; FRW, free-running wheel; HIIT, high-intensity interval training. Data are expressed as the mean ± SEM. Statistical significant differences (*p* ≤ 0.05) are indicated as follows: ^∗^ vs. SED, ^#^ vs. FRW, ^$^ vs. HIIT.

The FRW and HIIT exercise protocols elicited alterations in individual capillarization of the different fiber types (Figure 11). FRW increased the NCF of FOG fibers in the TA lateral (6.90 ± 0.30 vs. 5.30 ± 0.29 and 6.50 ± 0.38) and posterior fields (8.10 ± 0.43 vs. 5.80 ± 0.50 and 7.10 ± 0.37). Regarding the FIG and FG fibers, the FRW model induced an increase of NCF in the TA medial and posterior fields (Figure 11B,C), while in the lateral filed, it only increased the NCF of FG fibers (6.90 ± 0.30 vs. 5.30 ± 0.29 and 6.50 ± 0.38). Furthermore, HIIT increased the NCF of FIG and FG fibers of TA lateral and medial fields. However, in the TA posterior field, HIIT only increased the NCF of FG fibers (Figure 11A–C). Regarding the SOL muscle, both exercise models increased the NCF of SO fibers (10.90 ± 0.35 and 8.40 ± 0.29 vs. 6.70 ± 0.58). Furthermore, the FRW model also induced an increase in NCF of SO fibers comparing to HIIT (10.90 ± 0.35 vs. 8.40 ± 0.29). However, in the FOG fibers, HIIT induced an increase in NCF comparing to SED and FRW (10.10 ± 0.17 vs. 6.20 ± 0.63 and 5.00 ± 0.22). Additionally, the NCF of FOG fibers decreased in the FRW (Figure 11D).

Regarding CCA, FRW, and HIIT increased the CCA of FOG fibers in the TA medial and lateral fields, while only the HIIT regimen induced an increase of the FOG-related CCA in the TA posterior field (Figure 11E–G). Furthermore, both exercise models increased muscle capillarization of the SO fibers from SOL (Figure 11H).

### Capillary Density and Capillary-to-Fiber Ratio

**FIGURE 12:**
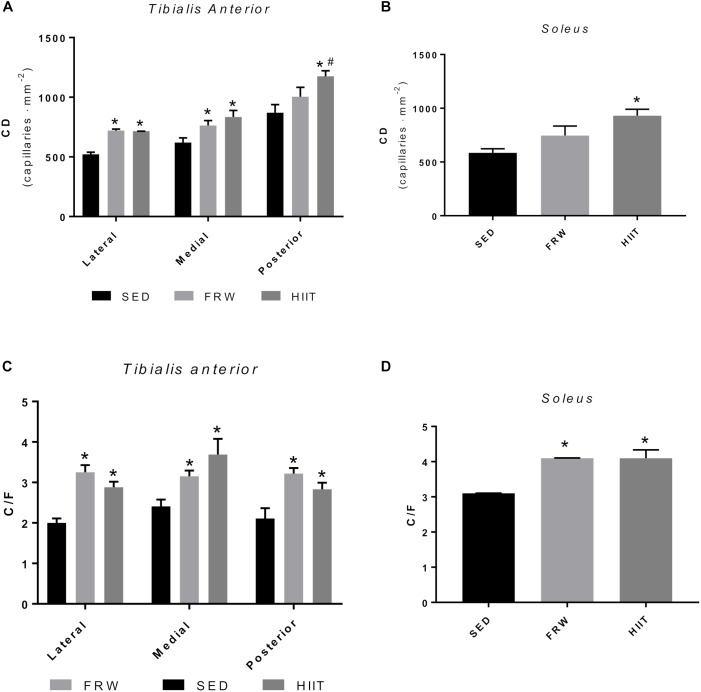
Effects of physical exercise on the capillary density (CD) **(A,B)**, and capillary-to-fiber ratio (C/F) **(C,D)** in three *tibialis* anterior regions and in *soleus* muscle. SED, sedentary; FRW, free-running wheel; HIIT, high-intensity interval training. Data are expressed as the mean ± SEM. Statistical significant differences (*p* ≤ 0.05) are indicated as follows: ^∗^ vs. SED, ^#^ vs. FRW.

Both exercise models increased CD in all the TA fields. Although this increase was not significant in the posterior region after FRW, the trend is clear (Figure 12A). The same trend for increasing was observed in the SOL muscle in the FRW group and a significantly greater CD after HIIT protocol was noted (Figure 12B).

Regarding C/F, the FRW, and HIIT models significantly increased this morphological feature in the three fields of the TA and in SOL (Figure 12C,D).

## Discussion

The overall findings of the present study suggest that 12 weeks of FRW, performed in a running wheel-equipped cage, as well as 12 weeks of forced training, using a HIIT protocol in a treadmill, induced similar physiological and biochemical adaptations at different levels of cellular organization in skeletal and cardiac muscles. From both central and peripheral point of view, FRW and HIIT regimens positively modulated animals’ functional capacity to perform maximal exercise. Specifically, both protocols induced alterations in the skeletal muscle fiber morphology and metabolism toward a more oxidative phenotype, as well as in cardiac mitochondria, which exhibited a more efficient energetic phenotype.

### Maximal Workload Tests

The FRW model has been used in rodents to attenuate the lack of physical activity associated with sedentary way of life (Fonseca et al., 2011a,b; Hyatt et al., 2015). In addition, it is also frequently used to avoid further impairments caused by lack of metabolic and mechanical stimuli or as a specific exercise regimen established to induce organic adaptations and thus, to positively modulate tissue phenotype (Kariya et al., 2004; Marques-Aleixo et al., 2016; Rocha-Rodrigues et al., 2016, 2017). In accordance with data from previous studies (Deloux et al., 2017; Stolle et al., 2017; Zhou et al., 2017), our results suggest that FRW induced significant adaptations regarding animals’ physical fitness. In fact, as previously mentioned, the adaptations produced by both exercise models were significantly different compared to the SED group along the different analyzed parameters. Our results showed that 12 weeks of HIIT, but also of FRW, induced an increase in the maximal speed evaluated in the MWT, a very important outcome related to animals’ functional capacity, even though the maximal speed achieved by HIIT-trained animals was higher than that of FRW. Maximal BLC decreased in both groups, with HIIT presenting lower levels than FRW. These two functional-related parameters are clear indicators of the increased animals’ ability to perform maximal exercise and of their metabolic response to high-levels of exercise through an improvement of the aerobic machinery at both central and peripheral levels. From an integrative metabolic perspective, it is possible that the altered skeletal muscle phenotype induced by both chronically intermittent exercise modalities, from more glycolytic into more oxidative-based fibers, might favor an increased efficiency of exercised groups in the lactate metabolization and transformation. This could have occurred through the known autocrine, paracrine, and endocrine links between glycolytic and oxidative metabolisms sustaining the complex mechanistic concept of lactate shuttle (Ferguson et al., 2018).

Alterations in rats’ T_tail_ have been related to exercise intensity (Wanner et al., 2015b). Actually, considering the lack of sweat response in rats, it is known that rats use the blood flow to the tail as a thermoregulatory mechanism (Wanner et al., 2015a). Accordingly, Tanaka et al. (1988) demonstrated that tail vasodilation increases in proportion to the exercise intensity at a range from 45 to 75% of the VO_2max_; however, at intensities higher than 75% of the VO_2max_, this positive linear association does not occur. Therefore, T_tail_ variations in response to similar metabolic activities imposed by treadmill exercise workloads during MWT were used as endpoints of the thermoregulatory response. The T_tail_ started to decrease in the early MWT low-intensity exercise stages and then rapidly increased probably due to skin vasodilatation mechanisms in order to contribute to heat release. This increase in the T_tail_ reached a plateau, which was sustained until the exhaustion (Figure 6). It is of note that, for both exercise protocols, the first significant differences between beginning MWT and end MWT in T_tail_ were observed at the same speed (36 cm ⋅ s^−1^). Moreover, both FRW and HIIT programs resulted in similar kinetics in T_tail_ curves during MWT. Thus, for both exercise protocols, the observed general T_tail_ increase during the 12th week MWT suggest similar thermoregulatory adaptations translated into an increased ability to lose heat by the tail during MWT.

### Alterations in Body Weight

The FRW group had higher body weight (in line with sedentary animals) than HIIT animals at the end of the 12 weeks, which can possibly be associated with different weight-related metabolic remodeling features induced by both exercise models. HIIT training has been suggested as a model of exercise that induce body weight reduction in humans, as it comprises high intensity bouts, with consequent increases in energy expenditure (Ramos-Filho et al., 2015; Kong et al., 2016) not only during the course of the exercise itself, but also in the subsequent period (Schaun et al., 2017). Considering that FRW animals run a significantly higher distance/day than their HIIT counterparts, the global exercise volume performed in the context of the present experimental design did not seem to have *per se* an effective role in the body weight decreases, which is in accordance with other works (Goncalves et al., 2015; Rocha-Rodrigues et al., 2016). Even though we did not measure it in the present study, an empirical assumption suggests that the higher exercise volume accomplished by the FRW rats, in contrast to the historically confirmed influence of continuous long lasting aerobic exercise on body weight (Ascensao et al., 2005), was performed through very short intermittent bouts interspersed by long recovery periods when compared to the 4 min bouts performed by the rats engaged in the HIIT program established in this study, which may compromise its effectivity on body weight alterations. Moreover, despite the methodological care regarding animals’ handling and environmental stress imposed in this study, we cannot exclude the possibility that, at least in part, an additional stress evoked by the forced HIIT program, when compared to FRW, may had influenced animals’ metabolism and eating and sleep habits with consequences on body weight alterations.

### Morphological and Metabolic Adaptations on Skeletal Muscle

The results were extremely consistent among the different morphometrical and capillarization evaluated parameters (fiber type distribution, fiber size and shape, muscle and fiber capillarization), as well as in the CS activity, suggesting a positive adaptation toward a more oxidative phenotype in both FRW and HIIT compared to the SED hypokinetic animals. In fact, TA and SOL muscles of exercised animals showed an increased percentage of more markedly oxidative fibers (Figure 9), such as FOG in the lateral and medial fields of TA and SO in the posterior field of TA and in SOL. These alterations in the aerobic fiber type percentage were consistent with a decrease in FCSA (Figure 10) of the aerobic fibers (FOG and SO), which has been described as hallmark of morphological muscle modifications under situations where a challenge for oxygen supply was overcome by decreasing cellular diffusion distance to mitochondria (Panisello et al., 2007). On the other hand, FG fibers from TA muscle adapted to the stimulus increasing cross-sectional area in FRW and HIIT groups, suggesting that both models simultaneously induce fiber type-specific adaptations aimed to reduce muscle fatigability while maintaining force production needed during short bouts of high intensity activity.

Moreover, as indicates the reduction in circularity, both exercise models induced elongated transversal profiles in SO fibers from SOL muscle, thus resulting in higher fiber perimeters. As it is well-known that there is a substantial proportion of mitochondria distributed in a subsarcolemmal position (Weibel, 1984), longer fiber perimeters will allow more mitochondria situated at shorter diffusion distances from the capillaries. This, as has been proposed in other animal models, could be seen as a fiber morphometrical modification to facilitate the oxygen supply to mitochondria in situations of high aerobic demand (Johnston, 1982; Torrella et al., 1998). All the previously described modifications were reinforced by an increased fiber capillarization (CCA) and global field capillarization (CD and C/F). Indeed, these results suggest that both FRW and HIIT protocols induced angiogenesis as reflected by higher C/F and NCF values. Considered a crucial biomarker of both mitochondrial content and an important indicator of tissue oxidative fitness, CS activity was determined in skeletal muscles, being its activity augmented in both exercise regimen groups, although only in SOL muscle showed statistically significant differences.

### Cardiac Mitochondrial Function

In order to explore the potential impact of both FRW and HIIT models on central-related training adaptations, cardiac muscle mitochondrial respiratory capacity, an important hallmark reflecting cardiac metabolism and function (Abel and Doenst, 2011), was evaluated. In accordance with our previous studies dealing with models of forced endurance exercise (Ascensao et al., 2005; Magalhaes et al., 2013), our results revealed that FRW and HIIT induced beneficial cardiac mitochondrial adaptations compatible with an increase in the metabolic coupling. Mitochondrial RCR is influenced by several metabolic oxidative features and is recognized as the best general measure of mitochondrial function in isolated mitochondria (Brand and Nicholls, 2011). In the present study, cardiac mitochondria from both FRW and HIIT animals energized with complex I-linked substrates showed higher RCR compared to SED, which suggests that the metabolic coupling between oxygen consumption and ATP synthesis was improved. Nevertheless, when substrates for complex II were used, these improvements were only observed in the HIIT group. Likewise, mitochondrial-related data also showed that HIIT, but not FRW, induced an increase in mitochondrial phosphorylation efficiency in ATP production as measured by ADP/O ratio. This data suggest that physiological central mechanisms were also positively modulated by both HIIT and FRW and certainly contributed, at least in part, for the improved ability to perform maximal exercise through a more toward oxidative phenotype.

## Conclusion

Data from our study clearly suggest that FRW is an exercise model that induces adaptations comparable, in most cases, with a forced HIIT regimen. In fact, functional and thermoregulatory adaptations, as well as significant alterations in biochemical and morphological phenotype in both skeletal and cardiac muscles were found in FRW-exercise animals when compared to those engaged in a HIIT protocol.

## Ethics Statement

This study was carried out in accordance with guidelines for Care and Use of Laboratory Animals in Research advised by Federation of European Laboratory Animal Science Associations (FELASA). The Ethics Committee of the Faculty of Sport, University of Porto, and the National Government Authority (Direção Geral de Alimentação e Veterinánia – N° 0421/000/000/2018) approved the experimental protocol.

## Author Contributions

JB, AA, JT, JS, and JM conceived and designed the research. JB, JA, ES-A, PF, JS, SR-R, and JM conducted sample collection, processing, and data collection. JB, GS, DR-R, and JT performed image analysis. JB, AA, JS, and JM composed the initial manuscript. JT, AA, and JM revised and approved the final draft.

## Conflict of Interest Statement

The authors declare that the research was conducted in the absence of any commercial or financial relationships that could be construed as a potential conflict of interest.

## References

[B1] Abel E. D. Doenst T. (2011). Mitochondrial adaptations to physiological vs. pathological cardiac hypertrophy. *Cardiovas. Res.* 90 234–242. 10.1093/cvr/cvr015 21257612 PMC3115280

[B2] Ascensao A. Magalhaes J. Soares J. M. Ferreira R. Neuparth M. J. Marques F. (2005). Moderate endurance training prevents doxorubicin-induced in vivo mitochondriopathy and reduces the development of cardiac apoptosis. *Am. J. Physiol. Heart Circ. Physiol.* 289 H722–H731. 10.1152/ajpheart.01249.2004 15792986

[B3] Ascensao A. Magalhaes J. Soares J. M. Ferreira R. Neuparth M. J. Marques F. (2006). Endurance training limits the functional alterations of rat heart mitochondria submitted to in vitro anoxia-reoxygenation. *Int. J. Cardiol.* 109 169–178. 10.1016/j.ijcard.2005.06.003 16019086

[B4] Bhattacharya S. K. Thakar J. H. Johnson P. L. Shanklin D. R. (1991). Isolation of skeletal muscle mitochondria from hamsters using an ionic medium containing ethylenediaminetetraacetic acid and nagarse. *Anal. Biochem.* 192 344–349. 1903610 10.1016/0003-2697(91)90546-6

[B5] Brand M. D. Nicholls D. G. (2011). Assessing mitochondrial dysfunction in cells. *Biochem. J.* 435(Pt 2), 297–312. 10.1042/bj20110162 21726199 PMC3076726

[B6] Brooke M. H. Kaiser K. K. (1970). Muscle fiber types: how many and what kind? *Arch. Neurol.* 23 369–379. 4248905 10.1001/archneur.1970.00480280083010

[B7] Deloux R. Vitiello D. Mougenot N. Noirez P. Li Z. Mericskay M. (2017). Voluntary exercise improves cardiac function and prevents cardiac remodeling in a mouse model of dilated cardiomyopathy. *Front. Physiol.* 8:899. 10.3389/fphys.2017.00899 29187823 PMC5694775

[B8] Delwing-de Lima D. Ulbricht A. Werlang-Coelho C. Delwing-Dal Magro D. Joaquim V. H. A. Salamaia E. M. (2017). Effects of two aerobic exercise training protocols on parameters of oxidative stress in the blood and liver of obese rats. *J. Physiol. Sci.* 68 699–706. 10.1007/s12576-017-0584-2 29222739 PMC10718027

[B9] Estabrook R. W. (1967). Mitochondrial respiratory control and the polarographic measurement of ADP: o ratios. *Methods Enzymol.* 10 41–47.

[B10] Ferguson B. S. Rogatzki M. J. Goodwin M. L. Kane D. A. Rightmire Z. Gladden L. B. (2018). Lactate metabolism: historical context, prior misinterpretations, and current understanding. *Eur. J. Appl. Physiol.* 118 691–728. 10.1007/s00421-017-3795-6 29322250

[B11] Fonseca H. Goncalves D. Figueiredo P. Mota M. P. Duarte J. A. (2011a). Lifelong sedentary behaviour and femur structure. *Int. J. Sports Med.* 32 344–352. 10.1055/s-0031-1271679 21380972

[B12] Fonseca H. Moreira-Goncalves D. Esteves J. L. Viriato N. Vaz M. Mota M. P. (2011b). Voluntary exercise has long-term in vivo protective effects on osteocyte viability and bone strength following ovariectomy. *Calcif. Tissue Int.* 88 443–454. 10.1007/s00223-011-9476-2 21416225

[B13] Fouces V. Torrella J. R. Palomeque J. Viscor G. (1993). A histochemical ATPase method for the demonstration of the muscle capillary network. *J. Histochem. Cytochem.* 41 283–289. 7678272 10.1177/41.2.7678272

[B14] Freitas D. A. Rocha-Vieira E. Soares B. A. Nonato L. F. Fonseca S. R. Martins J. B. (2018). High intensity interval training modulates hippocampal oxidative stress, BDNF and inflammatory mediators in rats. *Physiol. Behav.* 184 6–11. 10.1016/j.physbeh.2017.10.027 29111230

[B15] Gibala M. J. Jones A. M. (2013). Physiological and performance adaptations to high-intensity interval training. *Nestle Nutr. Inst. Workshop Ser.* 76 51–60. 10.1159/000350256 23899754

[B16] Goncalves I. O. Maciel E. Passos E. Torrella J. R. Rizo D. Viscor G. (2014). Exercise alters liver mitochondria phospholipidomic profile and mitochondrial activity in non-alcoholic steatohepatitis. *Int. J. Biochem. Cell Biol.* 54 163–173. 10.1016/j.biocel.2014.07.011 25063232

[B17] Goncalves I. O. Passos E. Diogo C. V. Rocha-Rodrigues S. Santos-Alves E. Oliveira P. J. (2016). Exercise mitigates mitochondrial permeability transition pore and quality control mechanisms alterations in nonalcoholic steatohepatitis. *Appl. Physiol. Nutr. Metab.* 41 298–306. 10.1139/apnm-2015-0470 26905378

[B18] Goncalves I. O. Passos E. Rocha-Rodrigues S. Torrella J. R. Rizo D. Santos-Alves E. (2015). Physical exercise antagonizes clinical and anatomical features characterizing lieber-decarli diet-induced obesity and related metabolic disorders. *Clin. Nutr.* 34 241–247. 10.1016/j.clnu.2014.03.010 24746977

[B19] Hyatt H. W. Toedebusch R. G. Ruegsegger G. Mobley C. B. Fox C. D. McGinnis G. R. (2015). Comparative adaptations in oxidative and glycolytic muscle fibers in a low voluntary wheel running rat model performing three levels of physical activity. *Physiol. Rep.* 3:e12619. 10.14814/phy2.12619 26603455 PMC4673647

[B20] Johnston I. A. (1982). Quantitative analyses of ultrastructure and vascularization of the slow muscle fibres of the anchovy. *Tissue Cell* 14 319–328. 7112537 10.1016/0040-8166(82)90030-1

[B21] Kariya F. Yamauchi H. Kobayashi K. Narusawa M. Nakahara Y. (2004). Effects of prolonged voluntary wheel-running on muscle structure and function in rat skeletal muscle. *Eur. J. Appl. Physiol.* 92 90–97. 10.1007/s00421-004-1061-1 15014999

[B22] Kong Z. Fan X. Sun S. Song L. Shi Q. Nie J. (2016). Comparison of high-intensity interval training and moderate-to-vigorous continuous training for cardiometabolic health and exercise enjoyment in obese young women: a randomized controlled trial. *PLoS One* 11:e0158589. 10.1371/journal.pone.0158589 27368057 PMC4930190

[B23] Kregel K. C. Allen D. L. Booth F. W. Fleshner M. R. Henriksen E. J. Musch T. (2006). Resource book for the design of animal exercise protocols. *Am. Physiol. Soc.* 20 374–377.

[B24] Lumini-Oliveira J. Magalhaes J. Pereira C. V. Moreira A. C. Oliveira P. J. Ascensao A. (2011). Endurance training reverts heart mitochondrial dysfunction, permeability transition and apoptotic signaling in long-term severe hyperglycemia. *Mitochondrion* 11 54–63. 10.1016/j.mito.2010.07.005 20654738

[B25] Magalhaes J. Falcao-Pires I. Goncalves I. O. Lumini-Oliveira J. Marques-Aleixo I. Dos Passos E. (2013). Synergistic impact of endurance training and intermittent hypobaric hypoxia on cardiac function and mitochondrial energetic and signaling. *Int. J. Cardiol.* 168 5363–5371. 10.1016/j.ijcard.2013.08.001 24012275

[B26] Magalhaes J. Goncalves I. O. Lumini-Oliveira J. Marques-Aleixo I. Passos E. Rocha-Rodrigues S. (2014). Modulation of cardiac mitochondrial permeability transition and apoptotic signaling by endurance training and intermittent hypobaric hypoxia. *Int. J. Cardiol.* 173 40–45. 10.1016/j.ijcard.2014.02.011 24602319

[B27] Marques-Aleixo I. Santos-Alves E. Balca M. M. Moreira P. I. Oliveira P. J. Magalhaes J. (2016). Physical exercise mitigates doxorubicin-induced brain cortex and cerebellum mitochondrial alterations and cellular quality control signaling. *Mitochondrion* 26 43–57. 10.1016/j.mito.2015.12.002 26678157

[B28] Nachlas M. M. Tsou K. C. De Souza E. Cheng C. S. Seligman A. M. (1957). Cytochemical demonstration of succinic dehydrogenase by the use of a new p-nitrophenyl substituted ditetrazole. *J. Histochem. Cytochem.* 5 420–436. 10.1177/5.4.420 13463314

[B29] Novak C. M. Burghardt P. R. Levine J. A. (2012). The use of a running wheel to measure activity in rodents: relationship to energy balance, general activity, and reward. *Neurosci. Biobehav. Rev.* 36 1001–1014. 10.1016/j.neubiorev.2011.12.012 22230703 PMC4455940

[B30] Panisello P. Torrella J. R. Pages T. Viscor G. (2007). Capillary supply and fiber morphometry in rat myocardium after intermittent exposure to hypobaric hypoxia. *High Alt. Med. Biol.* 8 322–330. 10.1089/ham.2007.1030 18081508

[B31] Ramos-Filho D. Chicaybam G. de-Souza-Ferreira E. Guerra Martinez C. Kurtenbach E. Casimiro-Lopes G. (2015). High intensity interval training (hiit) induces specific changes in respiration and electron leakage in the mitochondria of different rat skeletal muscles. *PLoS One* 10:e0131766. 10.1371/journal.pone.0131766 26121248 PMC4488295

[B32] Rocha-Rodrigues S. Rodriguez A. Becerril S. Ramirez B. Goncalves I. O. Beleza J. (2017). Physical exercise remodels visceral adipose tissue and mitochondrial lipid metabolism in rats fed a high-fat diet. *Clin. Exp. Pharmacol. Physiol.* 44 386–394. 10.1111/1440-1681.12706 27873387

[B33] Rocha-Rodrigues S. Rodriguez A. Gouveia A. M. Goncalves I. O. Becerril S. Ramirez B. (2016). Effects of physical exercise on myokines expression and brown adipose-like phenotype modulation in rats fed a high-fat diet. *Life Sci.* 165 100–108. 10.1016/j.lfs.2016.09.023 27693382

[B34] Schaun G. Z. Alberton C. L. Ribeiro D. O. Pinto S. S. (2017). Acute effects of high-intensity interval training and moderate-intensity continuous training sessions on cardiorespiratory parameters in healthy young men. *Eur. J. Appl. Physiol.* 117 1437–1444. 10.1007/s00421-017-3636-7 28488137

[B35] Shirvani H. Arabzadeh E. (2018). Metabolic cross-talk between skeletal muscle and adipose tissue in high-intensity interval training vs. moderate-intensity continuous training by regulation of PGC-1alpha. *Eat. Weight Disord.* 10.1007/s40519-018-0491-4 [Epub ahead of print]. 29480414

[B36] Songstad N. T. Kaspersen K. H. Hafstad A. D. Basnet P. Ytrehus K. Acharya G. (2015). Effects of high intensity interval training on pregnant rats, and the placenta. Heart and liver of their fetuses. *PLoS One* 10:e0143095. 10.1371/journal.pone.0143095 26566220 PMC4643918

[B37] Srere P. (1969). [1] Citrate synthase:[EC 4.1. 3.7. citrate oxaloacetate-lyase (CoA-acetylating)]. *Methods Enzymol.* 13 3–11.

[B38] Stolle S. Ciapaite J. Reijne A. C. Talarovicova A. Wolters J. C. Aguirre-Gamboa R. (2017). Running-wheel activity delays mitochondrial respiratory flux decline in aging mouse muscle via a post-transcriptional mechanism. *Aging Cell* 17:e12700. 10.1111/acel.12700 29120091 PMC5770778

[B39] Tanaka H. Yanase M. Nakayama T. (1988). Body temperature regulation in rats during exercise of various intensities at different ambient temperatures. *Jpn. J. Physiol.* 38 167–177. 3172577 10.2170/jjphysiol.38.167

[B40] Torrella J. Fouces V. Palomeque J. Viscor G. (1998). Comparative skeletal muscle fibre morphometry among wild birds with different locomotor behaviour. *The J. Anat.* 192 211–222. 9643422 10.1046/j.1469-7580.1998.19220211.x PMC1467755

[B41] Torrella J. R. Whitmore J. M. Casas M. Fouces V. Viscor G. (2000). Capillarity, fibre types and fibre morphometry in different sampling sites across and along the tibialis anterior muscle of the rat. *Cells Tissues Organs* 167 153–162. 10.1159/000016778 10971039

[B42] Van Norman K. H. (1909). The Biuret Reaction and the cold nitric acid test in the recognition of protein. *Biochem. J.* 4 127–135. 16742134 10.1042/bj0040127 PMC1276295

[B43] Wanner S. P. Leite L. H. Guimaraes J. B. Coimbra C. C. (2015a). Increased brain L-arginine availability facilitates cutaneous heat loss induced by running exercise. *Clin. Exp. Pharmacol. Physiol.* 42 609–616. 10.1111/1440-1681.12407 25881674

[B44] Wanner S. P. Prímola-Gomes T. N. Pires W. Guimarães J. B. Hudson A. S. R. Kunstetter A. C. (2015b). Thermoregulatory responses in exercising rats: methodological aspects and relevance to human physiology. *Temperature* 2 457–475. 10.1080/23328940.2015.1119615 27227066 PMC4844073

[B45] Weibel E. R. (1984). *The Pathway for Oxygen: Structure and Function in the Mammalian Respiratory System*. Cambridge, MA: Harvard University Press.

[B46] Zhou W. Barkow J. C. Freed C. R. (2017). Running wheel exercise reduces alpha-synuclein aggregation and improves motor and cognitive function in a transgenic mouse model of Parkinson’s disease. *PLoS One* 12:e0190160. 10.1371/journal.pone.0190160 29272304 PMC5741244

